# Estimating HIV incidence over a decade in Zimbabwe: A comparison of the catalytic and Farrington models

**DOI:** 10.1371/journal.pgph.0001717

**Published:** 2023-09-14

**Authors:** Rutendo Beauty Birri Makota, Eustasius Musenge

**Affiliations:** Division of Epidemiology and Biostatistics, School of Public Health, Faculty of Health Sciences, University of the Witwatersrand, Johannesburg, South Africa; McMaster University, CANADA

## Abstract

Over the years, numerous modelling studies have been proposed to estimate HIV incidence. As a result, this study aimed to evaluate two alternative methods for predicting HIV incidence in Zimbabwe between 2005 and 2015. We estimated HIV incidence from seroprevalence data using the catalytic and Farrington-2-parameter models. Data were obtained from 2005–06, 2010–11, and 2015 Zimbabwe Demographic Health Survey (ZDHS). These models were validated at the micro and macro-level using community-based cohort incidence and empirical estimates from UNAIDS EPP/SPECTRUM, respectively. The HIV incidence for the catalytic model was 0.32% (CI: 0.28%, 0.36%), 0.36% (CI: 0.33%, 0.39%), and 0.28% (CI: 0.26%, 0.30%), for the years 2005–06, 2010–11, and 2015, respectively. The HIV incidence for the Farrington model was 0.21% (CI: 0.16%, 0.26%), 0.22% (CI: 0.20%, 0.25%), and 0.19% (CI: 0.16%, 0.22%), for the years 2005–06, 2010–11, and 2015, respectively. According to these findings, the catalytic model estimated a higher HIV incidence rate than the Farrington model. Compared to cohort estimates, the estimates were within the observed 95% confidence interval, with 88% and 75% agreement for the catalytic and Farrington models, respectively. The limits of agreement observed in the Bland-Altman plot were narrow for all plots, indicating that our model estimates were comparable to cohort estimates. Compared to UNAIDS estimates, the catalytic model predicted a progressive increase in HIV incidence for males throughout all survey years. Without a doubt, HIV incidence declined with each subsequent survey year for all models. To improve programmatic and policy decisions in the national HIV response, we recommend the triangulation of multiple methods for incidence estimation and interpretation of results. Multiple estimating approaches should be considered to reduce uncertainty in the estimations from various models.

## Background

Understanding the dynamics of HIV epidemics is essential for determining and evaluating strategies for combating HIV [1]. The availability of antiretroviral therapy (ART) for people living with HIV (PLHIV) has significantly increased their lifespan, resulting in a larger population of HIV-positive individuals [2]. This shift from HIV as an acute condition to a chronic one highlights the importance of accurate estimates of HIV incidence and prevalence for effective public health responses [3]. Quantifying the dynamics of HIV epidemics is crucial for evaluating the efficacy of interventions and guiding policy to enhance HIV/AIDS control [1]. HIV prevalence and incidence are two primary indicators used to characterise the dynamics of the HIV epidemic. Obtaining accurate estimates of HIV incidence and prevalence has always been a significant health concern before the introduction of antiretroviral treatment (ART). Additionally, utilising HIV prevalence as an indicator of the epidemic has proved difficult due to differences in the time interval between infection and mortality due to increased ART availability [4]. To get a real-time evaluation of the HIV epidemic, HIV incidence estimation can provide greater insights [5]. Unfortunately, since HIV infection shares similar signs and symptoms with other diseases, individuals who have been recently infected with HIV may remain undiagnosed for significant periods, resulting in failing to detect the HIV incidence [1].

A significant number of modelling studies have been developed and proposed to predict HIV incidence [1, 4, 6, 7]. A few mathematical or statistical modelling studies have been developed in Zimbabwe to estimate HIV incidence. Among the few HIV incidence models done in Zimbabwe, Hargrove et al. used a simple Susceptible-Infected (SI) model to estimate the HIV incidence among perinatal women in Zimbabwe between 1990 and 2009 [8]. Gregson et al. used mortality data to estimate the HIV incidence in Zimbabwe using back-calculation methods [9]. Using population data from a survey done in 2015 under the Zimbabwe Population-Based HIV Impact Assessment Survey (ZIMPHIA 2015–2016), Gonese et al. compared the HIV incidence estimates obtained through recency testing to UNAIDS Spectrum incidence estimates [10]. Lastly, Ali et al. estimated HIV incidence among female sex workers (FSW) using HIV prevalence by age and number of years since started selling sex (YSSS) between 2011–2017 [11]. These methods have revolutionised how HIV incidence is estimated in various contexts and datasets worldwide [4]. However, few comparison studies to improve practical estimating settings have been reported [4, 12–14]. Therefore, the primary objective of this study was to employ estimation models that have been used for incidence estimation to predict HIV incidence using seroprevalence data.

Serial prevalence surveys have been proposed as a less expensive and invasive alternative to collecting longitudinal data on a cohort of people to determine disease incidence [15]. However, there are two significant disadvantages to this strategy. The first limitation is that any estimate based on serial prevalence data implicitly assumes disease irreversibility due to a lack of exact data on the duration of the condition. Because HIV is an incurable illness, the first limitation has no bearing on our study. The second limitation will be that there will be differential mortality between the infected and uninfected individuals.

Differential selection occurs when the likelihood of being selected varies between infected and uninfected individuals in a cross-sectional sample, resulting in a skewed incidence estimate. For example, when infected people have a higher mortality rate than uninfected people, differential selection occurs; as a result, diseased individuals are less likely to be included in a cross-sectional sample of the living population, resulting in an underestimate of disease incidence [16]. Given the possibility that the rate of mortality or survival after infection may be a factor in predicting incidence, our primary objective was to investigate two models in this study: the catalytic model, which assumes differential mortality, and the Farrington model, which assumes non-differential mortality, to determine if there are differences in the estimates of these models. The catalytic model has been used to estimate the incidence of HIV using seroprevalence data. In contrast, the Farrington model has been used to estimate the incidence of infectious diseases other than HIV. Hence, our secondary objective was to determine if the Farrington model behaved similarly to the catalytic model previously used to estimate HIV incidence. Lastly, we wanted to validate the estimates obtained from these models at micro and macro-level using community-based cohort incidence and empirical estimates from UNAIDS EPP/SPECTRUM, respectively.

The goal of this strategy was to triangulate results such that these two models may be utilised among the existing models that can be used to estimate HIV incidence using seroprevalence data from the Demographic and Health Survey (DHS). Data from three major household surveys in Zimbabwe from 2005 to 2015 was used to apply the models to ascertain the HIV incidence in the country.

## Methods

### Survey data

Incidence estimation methods in this paper used national household survey data from the Zimbabwe Demographic Health Survey (ZDHS) conducted in 2005–06 [17], 2010–11 [18] and 2015 [19]. The three surveys applied a two-stage random sampling design. The first stage involved selecting enumeration areas (EAs), which allowed specific weights to be assigned in the design. The second stage involved a random sampling of a fixed number of households [17–19]. The survey collected data from consenting individuals with age, sex, marital status, and HIV status. Data were collected in all households on females and males aged 0–49 and 0–54 years, respectively. In addition, blood samples were collected from consenting individuals. The finger prick method collected blot spots on filter paper and transported them to a laboratory for testing. Two ELISA tests were performed, with the second being a retest for 5–10% of the negative and all positive tests. A new ELISA or Western Blot test was performed if there were discordant results on the two ELISA tests [17–19].

### Estimation models

The catalytic and Farrington models were used to predict the HIV incidence in Zimbabwe. The HIV incidence was estimated using the ZDHS seroprevalence data in both models. The catalytic model simultaneously accounted for differential mortality, whereas the Farrington model assumed non-differential mortality, which was of interest in this analysis. The models are described in the sections that follow:

### Catalytic model

This paper adapted Mossong et al.’s [20] catalytic model to estimate age-dependent HIV incidence. The catalytic model is described fully elsewhere [9, 20]. A lognormal function was used to fit the incidence function (Eq 1), with one survival parameter and three incidence parameters [20] to age-specific HIV seroprevalence data from the 2005–06, 2010–11 and 2015 ZDHS of individuals aged 15–49 years. In fitting the lognormal function, an offset corresponding to the age of sexual debut was set at 12 years [20]. Homogeneity across time was assumed within the three-time periods considered in this paper, and the chance of survival following HIV infection was assumed to follow a Weibull distribution. The model assumed differential mortality, which was estimated simultaneously with the HIV incidence.

$$\lambda (a)=\left\{ \begin{array}{l} \;0\\ \frac{\theta }{\sqrt{2\pi \sigma }(a-12)}exp(-\frac{{\left(log(a-12)-\mu \right)}^{2}}{2\sigma^{2}}) \end{array}\right.\begin{array}{c} if\;a\leq 12\;years\\ if\;a>12\;years \end{array}$$ 1

Where: λ(a) is the incidence function with parameters mean μ and standard deviation σ, the scale of θ and an offset corresponding to the age of sexual debut fixed at 12 years.

### Farrington model

The Farrington model is a non-linear model that has been used to estimate the force-of-infection for many infectious diseases, including measles, mumps, and rubella [15]. The Farrington model is fully described elsewhere [21], but briefly, the model assumes that the force-of-infection increases to a peak in a linear trend and then declines exponentially [22]. Additionally, the model assumes that the Force-of-infection (FOI) begins at zero at birth and reaches a maximum at an age corresponding to the maximum transmission rate among susceptible and infected people. This results in the Farrington-3-parameter model represented in Eq 2:

$$\lambda (a)=(\alpha a-\gamma ){exp}^{\left(-\beta a\right)}+\gamma$$ 2

where; the age-specific Force-of-infection (FOI) is represented by λ(a), a is the age in years, and α, β and γ are model constants to be estimated with γ as the long-term residual value of the FOI.

If the residual value is set to zero (i.e. γ = 0, the Force-of-infection declines to zero as age tends to infinity (a ⟶ ∞), resulting in Eq 3, which is a Farrington-2-parameter model:

$$\lambda (a)=(\alpha a){exp}^{\left(-\beta a\right)}$$ 3

The Farrington model assumed non-differential mortality, which indicates that disease-related mortality did not affect how the observed serological profile was interpreted.

The age-specific incidence function, λ(a), in Eq 3 was estimated using age-specific seroprevalence data from the 2005–06, 2010–11, and 2015 ZDHS of persons aged 15–49 years.

### Validation of the Farrington and catalytic models

The Farrington and catalytic models were validated at micro and macro levels. The objective was to determine if our models could estimate HIV incidence at the provincial and national levels. At the micro-level, ZDHS data for Manicaland province was used to estimate the HIV incidence using the Farrington and catalytic models. Estimates obtained from these models were then compared to the observed incidence from a Manicaland Community-Based Cohort. Empirical estimates from the UNAIDS EPP/SPECTRUM were used to validate the Farrington and catalytic models at the national level.

### EPP/SPECTRUM model

UNAIDS created the Estimation Projection Package (EPP)/SPECTRUM [23–25] to estimate HIV prevalence and predictions [4]. The HIV prevalence estimated by EPP will then be used to estimate the HIV incidence using another software called SPECTRUM, which uses the AIDS Impact Model (AIM) module. A summary of the SPECTRUM methodology is as follows:

Let the number of HIV infections, the total number of adults in the population and the HIV prevalence of individuals aged a at time t be denoted by I_a,t_, N _a,t_ and P_a,t,_ respectively. Therefore;

$$I_{a,t}=N_{a,t}\times P_{a,t}$$ 4

and;

$$\lambda_{a,t}=I_{a,t}-(I_{a-1,t-1}-d_{a-1,t-1}^{A}-d_{a-1,t-1}^{NA})$$ 5

where;

λ_a,t_ is the number of new HIV infections in the year *t* minus the number of survived infections from the year t-1;

$d_{a-1,t-1}^{A}$ is the number of deaths caused by AIDS in year t-1; and

$d_{a-1,t-1}^{NA}$ is the number of deaths caused by other reasons other than AIDS in year t-1.

EPP was used to calibrate HIV prevalence data from household surveys using the maximum likelihood method, fitting a simple epidemiological model to surveillance data from prenatal clinics in Zimbabwe. Curves expressing the uncertainty in the prevalence data were created using Bayesian melding [26]. Finally, using assumptions about the average survival of HIV-infected people and the impact of ART on survival, adult HIV incidence was computed from HIV prevalence through time.

### Community-based cohort study–Manicaland

HIV incidence rates from community-based cohort studies in Zimbabwe from 2005 to 2015 were abstracted from published papers and conferences. Data from a community-based cohort study in Manicaland HIV/STD Prevention Project in Zimbabwe under the Alpha networks were used [27]. In addition, the Manicaland cohort HIV incidence data were taken from the supplemental material of research conducted by Hallet et al. [28]. The Manicaland cohort was a “closed cohort” in which those who joined within a certain time period, were not seen at a follow-up, or were not confirmed to have died were excluded.

### Ethical approval and consent to participate

This work was granted ethical clearance by the University of Witwatersrand’s Human Research Ethics Committee (Medical) (No. M151154). Application to the Measure DHS program, granted on May 16, 2017, yielded the dataset utilized in this investigation. The DHS Program is permitted to provide free survey data files to qualified academic researchers. Registration was required for access to data [17–19].

## Results

**Fig 1:**
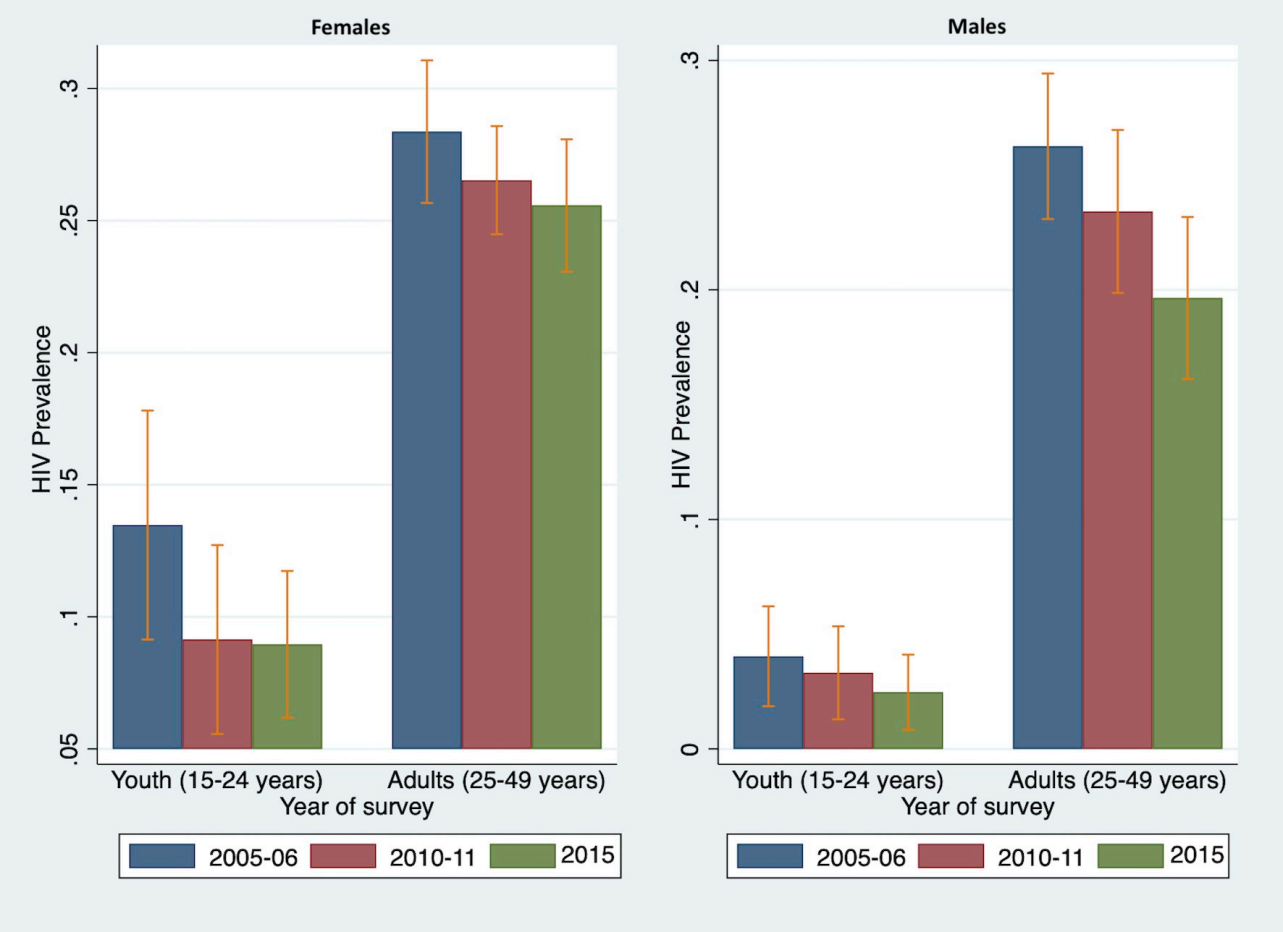
Prevalence trends by age group and survey year for females (left) and males (right).

The ZDHS prevalence data from 2005–06, 2010–11, and 2015 served as the primary input data to inform indirect model-based incidence estimates. Fig 1 displays the changes in HIV prevalence by age group, sex, and survey year. Generally, the HIV prevalence was higher in females than in males for both youth and adults. In addition, the HIV prevalence was higher for adults (25–49 years) than for youth (15–24 years) for both sexes. The HIV prevalence also declines with each successive survey year for both sexes.

### HIV incidence for the two models

Table 1 shows the annual HIV incidence for each modelling method stratified by sex. The HIV incidence for the catalytic model was 0.32% (CI: 0.28%, 0.36%), 0.35% (CI: 0.33%, 0.38%) and 0.28% (CI: 0.25%, 0.30%) for the year 2005–06, 2010–11, and 2015, respectively. The HIV incidence for the Farrington-2-parameter model was 0.21% (CI: 0.16%, 0.26%), 0.22% (CI: 0.20%, 0.25%) and 0.19% (CI: 0.16%, 0.22%) for the year 2005–06, 2010–11, and 2015, respectively. Based on these results, HIV incidence estimates were higher for the catalytic model than for the Farrington model. Table 1 also shows that HIV incidence increased from 2005–06 to 2010–11 and decreased in 2015 for both the catalytic and the Farrington-2-parameter models.

**Table 1:** Overall annual HIV incidence for the two models by year of survey.

	Catalytic	Farrington
Year	Incidence % (CI %)	Incidence % (CI %)
2005–06	0.32 (0.28,0.36)	0.21 (CI: 0.16, 0.26)
2010–11	0.36(0.33,0.39)	0.22(CI: 0.20, 0.25)
2015	0.28(0.26,0.30)	0.19(CI: 0.16, 0.22)

**Fig 2:**
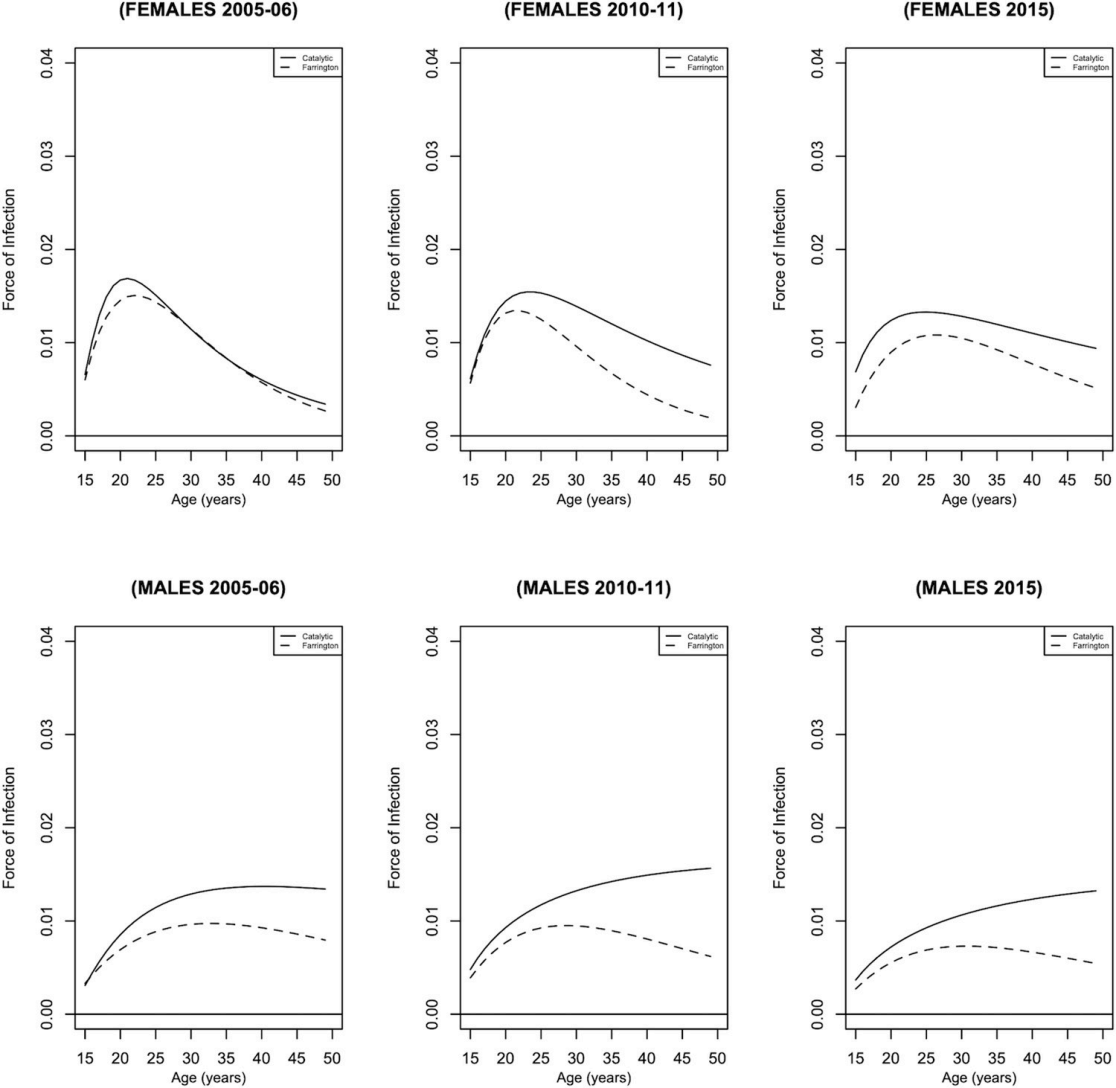
HIV incidence for males and females based on the catalytic and Farrington models.

As seen in Fig 2, the Farrington model underestimates HIV incidence compared to the catalytic model. Rather than the typical pattern observed in HIV incidence estimates for females, in which the incidence increases to a peak and then declines, HIV incidence estimates for males in 2010–11 and 2015 increased gradually without declining for the catalytic model. According to the Akaike Information Criteria (AIC), the catalytic model was the best fit for the survey data.

### Models’ validation using a cohort study

**Fig 3:**
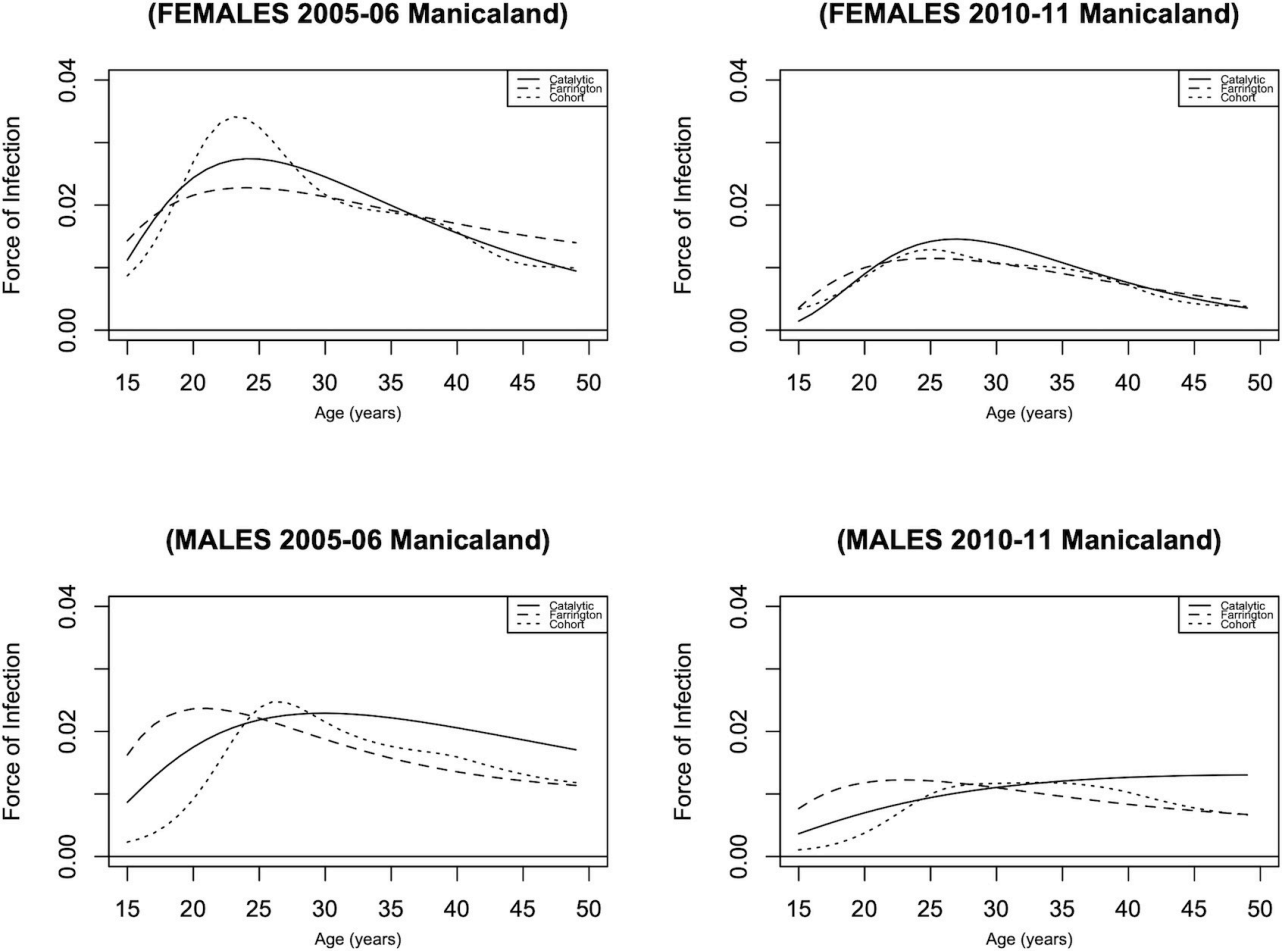
HIV incidence estimates based on the cohort, catalytic and Farrington models.

We obtained Manicaland cohort HIV measured incidence for the years 2005 and 2010. Therefore, comparisons were made based on these two available cohort years, as shown in Fig 3.

Table 2 summarises Manicaland’s estimated and observed incidence in various age groups. Almost all estimations fall within the observed measurements’ 95% confidence range. Both models performed well overall since the pattern and incidence are almost identical to the observed values, as shown in Table 2 and Fig 3. Table 2 shows that the estimations are within the 95% confidence interval of the observed, with 88% and 75% agreement scores for the catalytic and Farrington models.

**Table 2:** Measurements and estimates of incidence using Manicaland province data.

2005–06							
		COHORT		CATALYTIC		FARRINGTON	
Sex	Age Group, Years	Observed Incidence	95% CI^a^	Estimated Incidence	Agreement	Estimated Incidence	Agreement
**Females**	**15–24**	0.0232	0.0186, 0.0286	0.0218	*** ^b^	0.0201	* ^b^
	**25–49**	0.0179	0.0136, 0.0233	0.0184	*** ^b^	0.0184	*** ^b^
**Males**	**15–24**	0.0097	0.0071, 0.0129	0.0161	–	0.0219	–
	**25–49**	0.0176	0.0119, 0.0240	0.0209	** ^b^	0.0155	*** ^b^
**2010–11**							
**Females**	**15–24**	0.0079	0.0057, 0.0106	0.0079	*** ^b^	0.0088	*** ^b^
	**25–49**	0.0083	0.0057, 0.0114	0.0095	** ^b^	0.0082	*** ^b^
**Males**	**15–24**	0.004	0.0025, 0.0061	0.0061	* ^b^	0.0109	–
	**25–49**	0.0101	0.0063, 0.0149	0.0120	*** ^b^	0.0092	*** ^b^

^a^CI; confidence interval

^b^ Agreement between estimate and observed: absolute error values

***<10%

**<20%

*<30%

**Fig 4:**
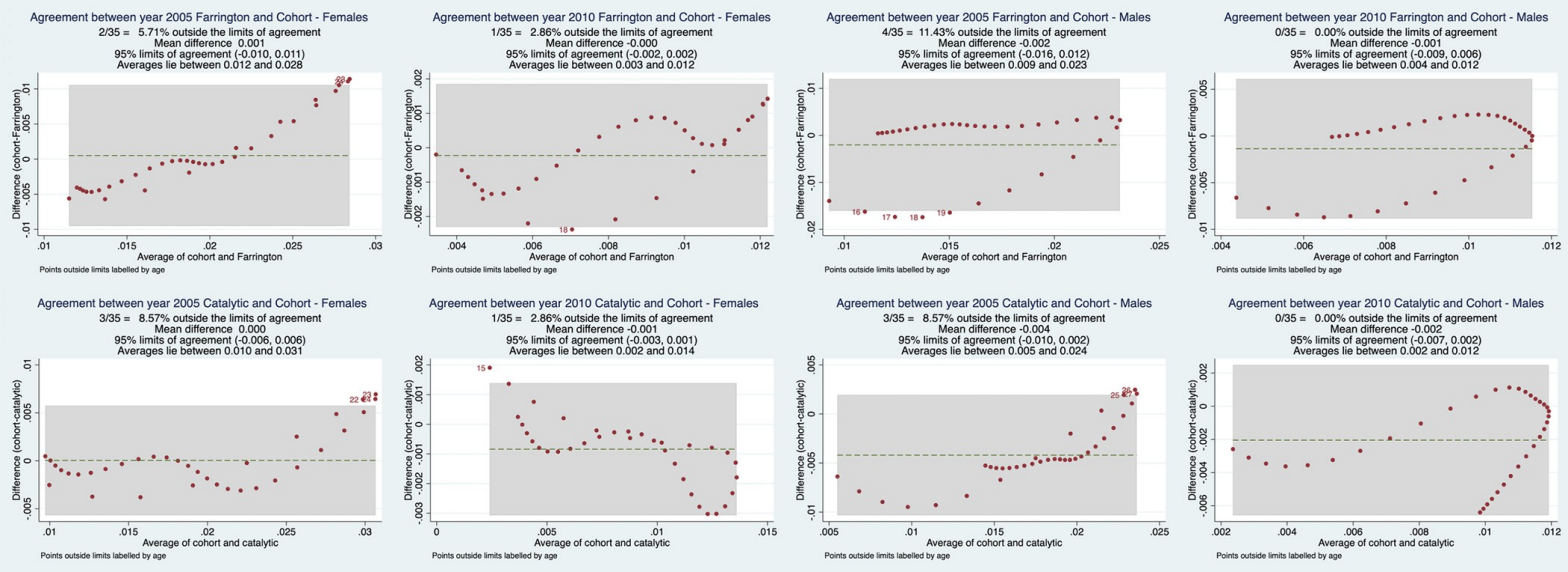
Bland-Altman plots demonstrating agreement between estimated incidence (Farrington and catalytic models) and observed incidence.

The agreement between observed (cohort) and estimated (Farrington/catalytic) incidence was assessed using Bland-Altman plots, as illustrated in Fig 4. The plots give two critical pieces of information: the average of all differences determined using the t-test and referred to as bias and the 95% limit of agreement. The limits of agreement shown in Fig 4 are narrow for all plots, indicating that our model estimates were comparable to cohort estimates. However, a few outlier values were observed in the 2005–06 Farrington females model, the 2010–11 catalytic females model, and the 2005–06 Farrington males model.

### Models validation using UNAIDS EPP estimates

**Fig 5:**
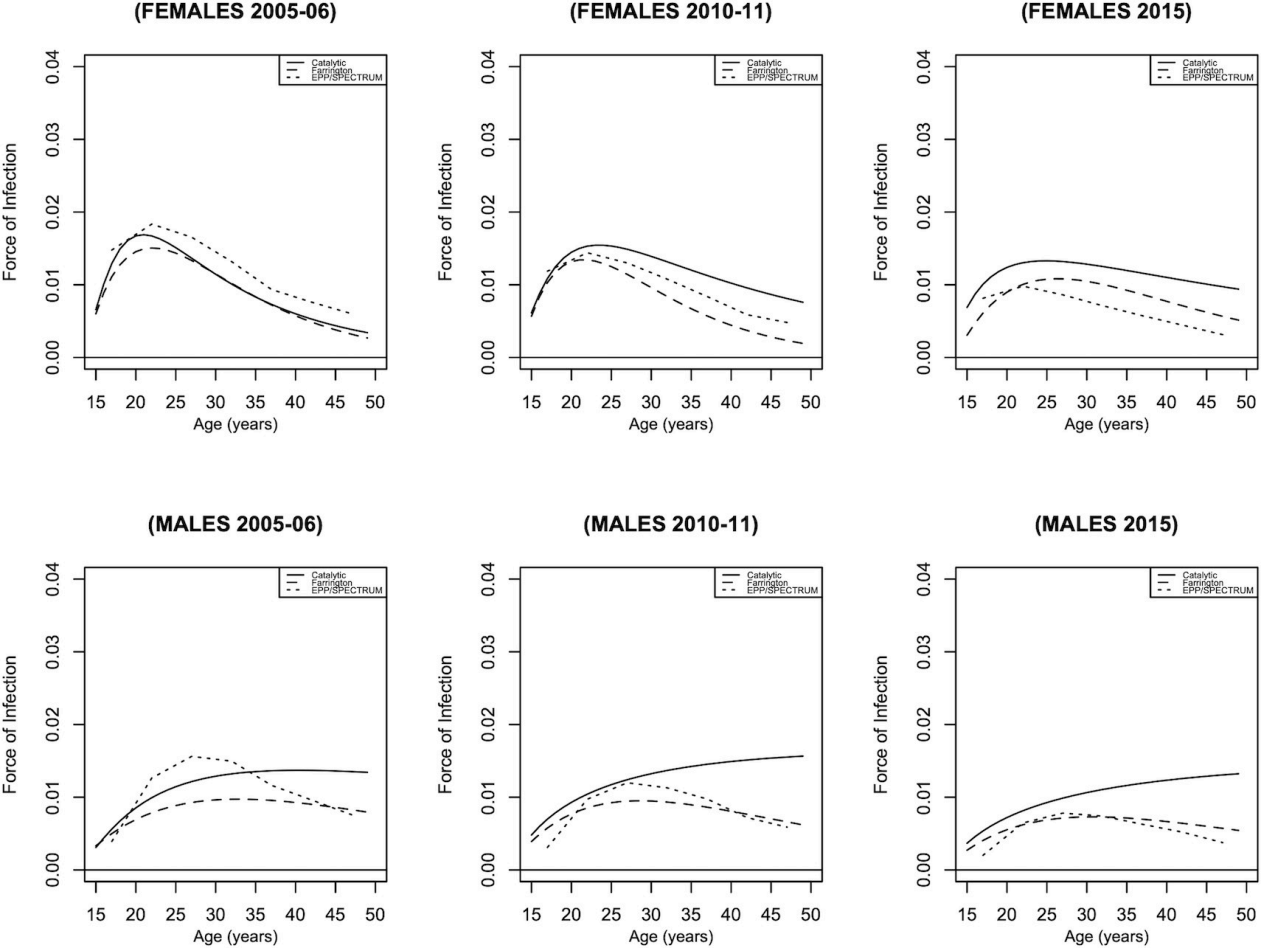
HIV incidence for males and females based on the catalytic, Farrington and EPP/SPECTRUM estimates.

Fig 5 illustrates the estimates of HIV incidence using three models: catalytic, Farrington, and EPP/SPECTRUM. From around 15 to 25 years, females had a higher HIV incidence than men; subsequently, the pattern reversed, with males having a higher HIV incidence. It is worth noting how the catalytic model predicts a progressive increase in HIV incidence for males throughout all survey years. HIV incidence declined with each subsequent survey year for all models. According to Fig 5, the Farrington model appeared more comparable to the UNAIDS EPP/SPECTRUM estimations.

## Discussion

Given the high expense of directly estimating HIV incidence using the “gold standard,” which requires long-term follow-up [29], indirect approaches based on mathematical or statistical methods have become cost-effective alternatives [4]. Therefore, we examined two models in this research that estimated age-specific HIV incidence using age-specific seroprevalence data. The objectives of this study were to estimate HIV incidence using the catalytic and Farrington models and to test the validity of these estimates at the national and sub-national levels.

The results indicate that HIV incidence estimates from the two models are substantially comparable in estimates and trends; nevertheless, caution should be exercised when interpreting the results due to the models’ different assumptions. The catalytic model best suited the data between the two models employed to predict HIV incidence, evidenced by its low AIC values. The Bland-Altman plots gave an approach to investigate the extent of agreement between the cohort HIV incidence estimates and the two models (catalytic and Farrington). Comparing the catalytic and Farrington models to the cohort observed estimates provides evidence that the two models can be used as part of the models that can be relied on in reporting the subnational HIV estimates in Zimbabwe. Overall, there was reasonable agreement between the two models and the cohort estimates, however, a few discrepancies were seen when some of the data points were not included in the 95% limits of agreement.

Although at the national level comparison, the trends of the two models seemed to be similar to the EPP/SPECTRUM trends, it should be noted that actual HIV estimates fluctuated over time. For example, we noticed that in 2005, the HIV estimates for the two models were underestimated as compared to the EPP/SPECTRUM model however, as we move with time, the inverse becomes true as the two models now overestimate the HIV incidence as compared to the EPP/SPECTRUM estimates. This may be accounted for by the changes that have been made to the EPP/SPECTRUM model over the years as a way to improve its estimates.

Despite considerable model assumption disparities, two mathematical models produced similar HIV incidence levels and trend estimates. Additionally, the models differed in their assumptions about death, with the catalytic model assuming differential mortality and the Farrington model assuming uniform mortality. As seen in Fig 2, the Farrington model generates lower HIV incidence estimates, possibly due to the differences in mortality assumptions; however, the differences seem minimal.

Estimating HIV incidence from serial age-specific cross-sectional prevalence data may be subject to several potential biases, including the quality of the age data, the comparability of the various survey populations, and changes over time in factors affecting HIV prevalence, such as socioeconomic status, migration, and fertility [30]. We believe that these possible biases are negligible in our investigation. This is also corroborated by research conducted by Batter et al. [30], who reported that the effect on mortality in their study was negligible. Other researchers, such as Bucyendore et al. [31] and Miotti et al. [32], utilized HIV prevalence data from surveys of childbearing women without adjusting for mortality or fertility impacts. Although mortality impacts of HIV infection are difficult to measure, they should ideally be included in HIV monitoring systems.

While estimating HIV incidence across age groups is challenging in the era of ART programs, it is critical for tracking changes in the age-specific pattern of HIV prevalence. Our findings imply that combining various approaches for estimating HIV incidence in the same population can aid in generating solid estimates of national HIV incidence levels and trends. This has been practised in many countries, for example, a study by Kim et al. [33] estimated HIV incidence among adults in Kenya and Uganda by comparing multiple methods, and they recommended that triangulation of methods was needed to determine the best supported estimates of incidence to guide programs. In Zimbabwe, Gonese et al. [10] validated the HIV incidence obtained through recency testing with the EPP/SPECTRUM estimates which are used in Zimbabwe by policymakers. In this study, unlike Gonese et al. [10] who used recency testing to estimate HIV incidence, we used statistical models to estimate HIV incidence.

To put the results of the two models into context, we note that young women between 2005 and 2015 had the highest risk of HIV incidence. However, our findings reveal a trend toward a decline in the HIV incidence of young women over time. Similarly, men over 30 years old are at an increased risk of HIV infection despite a gradual decline in recent years. Our findings corroborate the issue of age mixing in Zimbabwe [34, 35] and other sub-Saharan countries [36–39].

## Conclusion

Our study provides valuable insights into the trends in HIV prevalence and incidence among different age groups in Zimbabwe. The catalytic model outperformed the Farrington model in estimating HIV incidence, particularly in capturing the progressive increase in incidence observed in males. The observed agreement between the estimated and observed incidence values, as depicted in the Bland-Altman plots, further validates the reliability of our model-based estimates. However, a few outlier values suggest the need for caution when interpreting the results.

In conclusion, our findings contribute to the understanding of HIV dynamics in Zimbabwe and highlight the importance of utilising robust statistical models to estimate incidence and prevalence accurately. Further research is warranted to explore the underlying factors driving the observed differences in HIV incidence patterns between males and females.

Lastly, to improve programmatic and policy decisions in the national HIV response, we recommend the triangulation of multiple methods for incidence estimation and interpretation of results. Multiple estimating approaches should be considered to reduce uncertainty in the estimations from various models.
